# Value of regular endosonography and [^18^F]fluorodeoxyglucose PET–CT after surgery for gastro-oesophageal junction, stomach or pancreatic cancer

**DOI:** 10.1093/bjsopen/zraa028

**Published:** 2020-12-23

**Authors:** O S Bjerring, S Hess, H Petersen, C W Fristrup, L Lundell, M B Mortensen

**Affiliations:** Department of Surgery, Odense University Hospital, Odense, Denmark; OPAC, Odense Pancreas Centre, Odense University Hospital, Odense, Denmark; Department of Physiology and Nuclear Medicine, Odense University Hospital, Odense, Denmark; Department of Radiology and Nuclear Medicine, Hospital South West Jutland, Esbjerg, Denmark; Department of Regional Health Research, Faculty of Health Sciences, University of Southern Denmark, Odense, Denmark; Department of Physiology and Nuclear Medicine, Odense University Hospital, Odense, Denmark; Department of Surgery, Odense University Hospital, Odense, Denmark; OPAC, Odense Pancreas Centre, Odense University Hospital, Odense, Denmark; Department of Surgery, Odense University Hospital, Odense, Denmark; CLINTEC, Karolinska Institutet, Stockholm, Sweden; Department of Surgery, Odense University Hospital, Odense, Denmark; OPAC, Odense Pancreas Centre, Odense University Hospital, Odense, Denmark

## Abstract

**Background:**

Most patients undergo follow-up after surgery for cancers of the gastro-oesophageal junction, stomach or pancreas, but data to support which modalities to use and the frequency of investigation are limited.

**Methods:**

Patients in the EUFURO study were randomized to either visits to the outpatient clinic at 3, 6, 9, 12, 18, and 24 months after surgery (standard), or to the addition of [^18^F]fluorodeoxyglucose (FDG) PET–CT and endoscopic ultrasonography (EUS) with guided fine-needle aspiration biopsy to clinical assessments (intervention). Data from the intervention arm were used to analyse the diagnostic performance of endosonography or [18F]FDG PET–CT in detecting recurrences.

**Results:**

During the scheduled follow-up, 42 of 89 patients developed recurrence; PET–CT and EUS in combination detected 38 of these recurrences. EUS detected 23 of the 42 patients with recurrent disease during follow-up and correctly diagnosed 17 of 19 locoregional recurrences. EUS was able to detect isolated locoregional recurrence in 11 of 13 patients. In five patients, EUS was false-positive for isolated locoregional recurrence owing to missed distant metastases. PET–CT detected locoregional recurrence in only 12 of 19 patients, and isolated locoregional recurrence in only 7 of 13. False-positive PET–CT results in 23 patients led to a total of 44 futile procedures.

**Conclusion:**

Accuracy in detecting recurrences by concomitant use of PET–CT and EUS was high (90 per cent). PET–CT had moderate to high sensitivity for overall recurrence detection, but low specificity. EUS was superior to PET–CT in the detection of locoregional and isolated locoregional recurrences.

## Teaser

Endosonography and [^18^F]fluorodeoxyglucose PET–CT combined is highly accurate in detecting recurrent disease after radical operation for gastro-oesophageal, stomach or pancreatic cancers. PET–CT has a moderate to high sensitivity, but a low specificity, ultimately leading to a substantial number of additional and often futile examinations. Endosonography is highly sensitive and specific in detecting locoregional recurrences, and superior to PET–CT in detecting isolated locoregional recurrences, enabling potentially curative treatment. FP, false-positive; TP, true-positive.

## Introduction

Despite efforts to select patients presenting with gastro-oesophageal junction (GOJ), gastric, and pancreatic cancers for curative therapy, survival remains disappointing even after radical resection^1–7^. These malignancies carry a high risk of recurrent disease usually developing within 2 years of surgery^1^^,^^3^^,^^4^^,^^6^. By the time patients develop symptoms of recurrent disease, the disease burden is often sufficient to preclude further treatment that will extend survival significantly^3^. Strategies have been explored to detect recurrence at a stage when further therapies can be reasonably employed. In practice, most patients undergo postoperative follow-up, the duration and content of which vary substantially, largely reflecting lack of scientific evidence for its value^4^^,^^8–10^. A pivotal component of any follow-up protocol is access to sensitive and specific methods for the detection of recurrent disease at a time when novel treatment strategies are most likely to result in benefit to the patient.

Cross-sectional imaging, mainly CT or PET–CT, has been used widely in clinical practice and research protocols. These techniques are considered the standard of care for the detection of recurrent and/or metastatic disease, but may be unable to detect small isolated locoregional relapses, as the sensitivity for subcentimetre lesions is low^11^^,^^12^. The potential clinical gain from postoperative screening with CT or PET–CT may be limited to detecting disseminated recurrences amenable to palliative treatment only, although in recent years preliminary data have encouraged further studies of PET–CT in detection of local recurrence^13–17^.

Endoscopic ultrasonography (EUS) has been used widely in the pretherapeutic stratification of patients with GOJ, stomach, and pancreatic cancer. EUS with guided fine-needle aspiration biopsy (FNA) is recognized as an essential element in preoperative staging and resectability assessment^8–10^^,^^18^. In the postoperative situation, EUS has been alleged to detect recurrent disease in up to 50 per cent of patients, even in those without symptoms^13^^,^^19^. The obvious limitation of EUS is that it can only detect disease within the native organ or nearby, depending on the ultrasound frequency. EUS cannot detect distant metastatic lesions and must be supplemented by cross-sectional imaging to identify distant recurrences. The rationale behind many follow-up programmes is that early and asymptomatic recurrences are worth detecting, as potentially curative treatment might be offered, or that additional treatment might be more effective for a smaller disease burden. The relevant question remains: how accurate are imaging modalities regarding this specified target? A side study of the phase 2 EUFURO RCT, which compared two different follow-up strategies after curative treatment of upper gastrointestinal (GI) malignancies^3^, has therefore investigated the diagnostic accuracies in detecting recurrences overall by EUS or PET–CT, with emphasis on detection of isolated locoregional recurrence.

## Methods

All patients who had undergone radical resection for adenocarcinomas in the GOJ, stomach or pancreas in the Department of Surgery, Odense University Hospital, Denmark, and who were eligible for oncological treatment at the time of assessment 1 month after surgery, were invited to participate in the study. After informed written consent had been obtained, patients were randomized, by a computerized method (https://www.randomization.com/) whereby group allocation labels were placed in non-transparent envelopes. The randomization sequence was unknown to those responsible for patient enrolment in the outpatient clinical follow-up. Patients were randomized consecutively in the order in which they were referred for treatment. They were allocated to either standard follow-up, consisting of visits to the outpatient clinic at 3, 6, 9, 12, 18, and 24 months after operation, or intervention, comprising [^18^F]fluorodeoxyglucose (FDG) PET–CT and EUS + FNA added to the clinical assessment at the same time points. Only the patients in the intervention arm were investigated in the present study.

All patients were given written and oral information before entering the study. The study was approved by the Danish National Ethics Committee (S-20110004), and registered with the Danish Data Protection Agency (2014-41-3630) and at ClinicalTrials.gov (NCT02209415).

### PET–CT

Patients fasted for at least 6 h before FDG PET–CT^11^. FDG was administered intravenously according to patient weight with a dose of 4 MBq/kg (108 μCi/kg), minimum 200 MBq and maximum 400 MBq. After injection, the patient rested for 30 min after which they were hydrated with 800 ml water orally over less than 30 min. Some 60 (± 5) min after tracer injection, the patient was scanned from the base of the skull to the proximal femur. The first and last scans (after 3 and 24 months respectively) were performed with diagnostic contrast-enhanced CT to establish the postoperative anatomy, whereas the interim scans comprised low-dose CT without contrast, to reduce the radiation load. CT and PET were performed on either Discovery STE^™^, Discovery VCT, Discovery RX, Discovery 690 or Discovery 710 scanners (GE Medical Systems, Milwaukee, Wisconsin USA). PET–CT images were interpreted by specialists in nuclear medicine and radiology as part of daily routine practice.

### Endoscopic ultrasonography and fine-needle aspiration biopsy

EUS and EUS–FNA were performed under conscious sedation. A curvilinear array echoendoscope (FG-38UX; Pentax, Hamburg, Germany) attached to a Hi-vision Preirus scanner (Hitachi Medical Systems, Zug, Switzerland) were used. For the EUS–FNA procedures, either 19- or 22-G standard needles (Expect^™^, Boston Scientific, Boston, Massachusetts, USA; or Echotip^®^ Ultra, Cook, Limerick, Ireland) were used. No antibiotics were given. All endoscopists were highly experienced in upper GI EUS. For any lesion detected and considered as suspicious for recurrence on EUS, FNA was attempted according to departmental standards. Only if FNA confirmed recurrence, was the case brought forward to the multidisciplinary tumour (MDT) board.

### Data acquisition

Data from patient records regarding perioperative results were collected on case record forms. Time of recurrence was defined as the date of the conclusive MDT board meeting. Based on the outcome of the MDT board decision, both PET–CT and EUS procedures could be classified as either true-negative, true-positive, false-negative or false-positive, and the diagnostic performance of each procedure was calculated. If diagnostic findings had been classified as false-positive in the first instance and false-negative based on a later scan, this situation was categorized as false-negative in the final analysis, as this was considered to have the greatest clinical impact for the patient. If recurrence developed after the predefined follow-up of 24 months, the site of recurrence was compared with previous imaging results for findings that had been deemed false-positive at the same anatomical location.

All suspicious or confirmed lesions were presented and evaluated at the MDT board meeting. At least one surgical specialist, one medical oncologist, one radiologist, one nuclear medicine specialist. and a pathologist all with expertise in upper GI cancers participated in each meeting. The composite decision reached by the board was individualized for patient case. When a finding at the final PET–CT or EUS at 24 months was deemed false-positive by the MDT board, a follow-up scan was scheduled 3 months later.

Locoregional recurrences were defined as recurrences in the remnant of the resected organs or organs anastomosed to it, the resection bed, or locoregional lymph nodes, according to the seventh edition of the UICC classification^20^. Isolated locoregional recurrences were diagnosed when there were no signs of recurrent disease elsewhere. Recurrences in the liver, lungs, pleura, peritoneum, neck, bones, and lymph nodes, regarded as M1 according to seventh edition of the UICC staging system, were classified as distant metastases.

### Statistical analysis

Fisher’s exact test was used for comparison of categorical data between groups. Statistical analysis was carried out using Stata^®^ version 14.0 (StataCorp, College Station, Texas, USA).

## Results

**Fig. 1:**
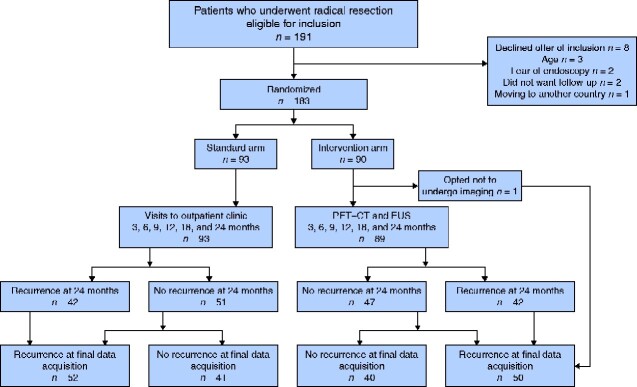
Flow chart illustrating management of patients enrolled in study For the present article, only patients in the intervention arm were analysed. EUS, endoscopic ultrasonography.

A total of 191 patients were invited to participate over a 3-year period, and 183 patients were enrolled (*Fig. 1*). Three patients were considered unfit (aged 85–89 years), two refused follow-up, two declined repeat endoscopies, and one patient moved abroad. Ninety patients were allocated to repeat EUS + PET–CT investigations, 30 women and one man, with a mean age of 63 (95 per cent c.i. 61 to 65) years. Tumour staging in relation to tumour location is shown in *Table 1*. One patient opted not to participate in the imaging procedures.

**Table 1:** Tumour charactistics in 90 patients randomized to the intervention arm

	**GOJ** **(*n* = 29)**	**Stomach** **(*n* = 22)**	**Pancreas** **(*n* = 39)**
**Tumour category**			
pT0	5	3	–
pT1	2	7	3
pT2	6	1	11
pT3	15	9	22
pT4	1	2	3
**Node category**			
pN0	18	14	18
pN1	7	4	21
pN2	3	2	–
pN3	1	2	–

Values in parentheses are percentages.

*After being randomized, one patient opted not to undergo endoscopic ultrasonography and and PET–CT. Tumours were staged according to the seventh edition of the UICC classification^20^. GOJ, gastro-oesophageal junction.

The remaining 89 patients underwent 383 scheduled EUS and PET–CT procedures. No adverse events were noted. None of the patients left the study before the protocol had ended or recurrence was detected.

### Detection of recurrence

During follow-up, 42 patients developed recurrence; PET–CT and EUS in combination detected 38 of these. The location of recurrences is summarized in *Table 2*. The remaining four patients developed symptoms of recurrence in between the planned scans, and were diagnosed accordingly.

**Table 2:** Recurrence site at time of detection

	**GOJ** **(*n* = 29)**	**Stomach** **(*n* = 22)**	**Pancreas** **(*n* = 38)**	**Total** **(*n* = 89)**
Oesophagus	2 (7)	2 (9)	0	4 (4)
Mediastinum	8 (28)	1 (5)	1 (3)	10 (11)
Lung	3 (10)	1 (5)	4 (11)	8 (9)
Pleura	4 (14)	0 (0)	1 (3)	5 (6)
Liver	3 (10)	2 (9)	12 (32)	17 (19)
Stomach	3 (10)	1 (5)	0 (0)	4 (4)
Pancreas	0 (0)	0 (0)	6 (16)	6 (7)
Bone	2 (7)	1 (5)	1 (3)	4 (4)
Retroperitoneum	4 (14)	2 (9)	7 (18)	13 (15)
Peritoneum	4 (14)	4 (18)	8 (21)	16 (18)
Neck	0 (0)	1 (5)	1 (3)	2 (2)
Abdominal wall	1 (3)	0 (0)	0 (0)	1 (1)
Cerebrum	1 (3)	0 (0)	0 (0)	1 (1)

Values in parentheses are percentages. GOJ, gastro-oesophageal junction.

### Endoscopic ultrasonography

EUS detected recurrent disease in 23 of 42 patients during follow-up and correctly diagnosed 17 of 19 locoregional recurrences. It detected isolated locoregional recurrence in 11 of 13 patients (*Table 3*). The specificity of EUS in detecting overall and local recurrence was by definition 100 per cent as cytomorphological confirmation through EUS–FNA was mandated by the protocol. In five patients EUS was false-positive for isolated locoregional recurrence, as distant metastases that occurred at the same time were not detected by EUS. The total number of FNA procedures was 88, of which 13 were repeat aspirations. Of these 13 repeat procedures, seven were negative and six confirmed recurrence. No adverse events occurred in connection with these procedures.

**Table 3:** Diagnostic performance of endoscopic ultrasonography and PET–CT for recurrence overall, locoregional recurrence and isolated local recurrence at 24 months after curative resection for upper gastrointestinal malignancies

	EUS			PET–CT		
	Recurrence, overall	**Locoregional** **recurrence**	Isolated local recurrence	Recurrence, overall	Locoregional recurrence	Isolated local recurrence
Sensitivity (%)	55	89	85	79	53	54
Specificity (%)	100	100	93	51	86	80
PPV (%)	100	100	69	59	55	32
NPV (%)	71	93	97	73	90	91
Accuracy (%)	79	98	92	64	70	86

EUS, endoscopic ultrasonography; PPV, positive predictive value; NPV, negative predictive value.

### [^18^F]FDG PET–CT

**Fig. 2:**
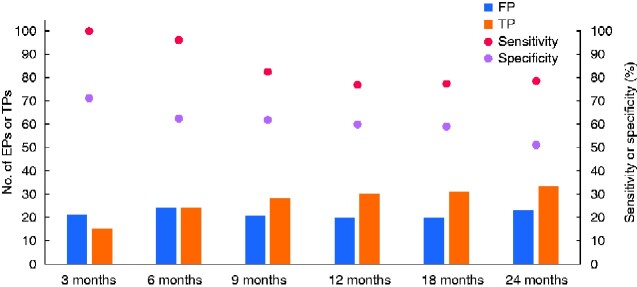
Cumulative diagnostic performance of PET–CT over time for 89 patients undergoing follow-up after curative surgery for upper gastrointestinal malignancies FP, false-positive; TP, true-positive.

Overall PET–CT detected 33 of the 42 recurrences that emerging during follow-up, and had superior sensitivity to EUS in the overall detection of recurrent disease (*P* = 0.037). The overall diagnostic performance of PET–CT is shown in *Table 3*. There was a steady decline in sensitivity and specificity over 24 months (*Fig. 2*). The true-positives, however, increased from 17 (19%) at 3 months to 37 (41%) at the end of the study. Overall, there was no significant variation in the performance of PET–CT between the three malignancies studied (*Fig. S1*). There was no statistically significant difference in diagnostic performance between high- and low-dose PET–CT (*Fig. 2*). PET–CT detected locoregional recurrence in 12 of 19 patients, and isolated locoregional recurrence in 7 of 13.

False-positive results in 23 patients resulted in a total of 44 futile examinations and procedures; the distribution of these procedures is listed in *Table S1*. No adverse events occurred in connection with these procedures. Of 23 patients with a false-positive result on PET–CT, five went on to develop recurrent disease after the scheduled follow-up. In only one of these patients was the site of recurrence (peritoneum) the same as that suspected based on the original PET–CT imaging. The first postoperative PET–CT at 3 months was positive, but there were no signs of recurrent disease on the following five PET–CT scans. The interval between the false-positive PET–CT and the final clinical diagnosis of recurrence was 29 months.

## Discussion

Recurrence is common after treatment designed to achieve cure in patients with upper GI malignancies. In the EUFURO study^3^, 102 of 183 patients overall relapsed during the relatively short scheduled follow-up of 24 months. In the present analysis, the overall ability of concomitant use of PET–CT and EUS detect recurrences was 90 per cent. EUS alone detected recurrence in just over half of those proven to have recurrent disease, and correctly diagnosed 17 of 19 locoregional recurrences. Isolated locoregional recurrences were detected by EUS in 11 of 13 patients, but at the expense of five false-positives where coincident distant metastases were not detected. Overall, PET–CT detected 33 of the 42 recurrences, but was hampered by poor detection of isolated local recurrence, reflected by a sensitivity of 54 per cent and a positive predictive value of only 32 per cent.

A recurring methodological shortcoming in this area is definition of the reference standard for detection of recurrent disease. The present study applied the standard used in a routine clinical setting. Composite findings were discussed by the MDT board, and scrutinized to establish the presence or absence of recurrent disease as an aggregate decision on how to proceed in the clinical management of each patient. According to the protocol, the study aimed for histopathological verification through biopsy; if this was not possible, follow-up scans were mandated.

As expected, EUS had low diagnostic sensitivity, owing to its inability to detect distant metastasis in most patients. This finding should not be confused with the fact that EUS-guided FNA has high accuracy when metastatic lesions such as those in the liver, resection bed, local lymph nodes, ascites or pleural effusions lie within reach of the EUS-guided needle^14^. The overall accuracy of 79 per cent for EUS reflected cytomorphological verification. The sensitivity of PET–CT was higher, but the significant number of false-positive findings reduced specificity and the overall diagnostic performance of this modality. This contrasts with some publications, where higher diagnostic accuracy with PET–CT was reported, with pooled sensitivities and specificities as high as 96 and 78 per cent respectively^15–17^^,^^21^, whereas others^22^^,^^23^ documented much lower figures that corroborate the findings of the present study.

In a study^24^ comparing PET–CT with endoscopy, CT and abdominal ultrasound imaging in a follow-up programme after surgery for oesophageal cancer, the sensitivity of PET–CT was 100 per cent with a specificity of 86 per cent. Most patients had squamous cell cancer and the recurrence rate was surprisingly high (70 per cent within the first year). This contrasts with the predominance of adenocarcinomas among patients with junctional cancers in the present study. The use of PET in the staging of, and search for, relapses of squamous cell cancers is well established^25^, unlike the situation for adenocarcinomas of the GOJ and stomach, where biological differences in glucose transporter-1 distribution^26^ may lead to substantial variability in FDG uptake. Low FDG accumulation is also recognized in pancreatic cancers^27^.

Other factors may also have contributed to the different results between studies. Some studies^28^^,^^29^ reported a high diagnostic performance of PET–CT, but examined patients with a high probability of harbouring recurrent disease. Details of the target populations are essential not only when they are followed up according to a predefined protocol, but also if they represent a cohort with a low, moderate or high risk of recurrent disease. Other studies^28–31^ have recruited patients presenting with symptoms suggestive of, or findings suspicious for, recurrent disease, thereby increasing the pretest probability. PET–CT studies often focus solely on the detection of distant metastases, whereas the present study, given the published survival data^3^, was undertaken to identify isolated locoregional recurrence where there might be the opportunity for further curative treatment.

From the survival data for these patients previously published by the present study group^3^, it is still questionable what, if any, impact the detection of early disseminated recurrences has on overall survival. With currently available treatments, overall survival does not change as a function of the follow up-strategy, but a difference has been observed in the selected group with isolated local recurrence^3^. In the present study, isolated locoregional recurrence was found in 13 of 42 patients (31 per cent) who were regularly followed up with EUS and PET–CT, a figure slightly higher than the 23 per cent reported elsewhere^6^, probably related to the more frequent examinations in the present study. In a recent study of oesophageal and GOJ cancers^32^, in which patients underwent imaging only if recurrence was suspected clinically, isolated local recurrence was found in only 10 per cent. It may be that this pattern of recurrence is not necessarily symptomatic and the time window for its detection can easily be missed. In the present study, EUS proved to be the most accurate modality for detecting isolated locoregional recurrence. The value of PET–CT in this context remains questionable. The clinical significance of these observations rests on the fact that patients with isolated local recurrences may have a better prognosis because more aggressive multimodal therapies, including further resections, can be offered^3^^,^^33^^,^^34^. Survival benefits for such strategies are limited, and patient selection remains a crucial factor behind a successful outcome. Patients treated at this centre with isolated locoregional recurrence survived significantly longer than those with disseminated recurrences^3^.

As is the case in all clinical trials, this study has limitations. The population investigated comprised a mixture of different disease entities, which hampers firm conclusions. This decision was based on a tradition of grouping these diseases according to diagnostic work-up^18^^,^^35^^,^^36^. The rarity of these diseases means that, even for a high-volume centre, the recruitment period for patients who underwent curative resection within each disease entity would be unfeasibly long, with the introduction of potential biases as other factors change over time. Even though the added value of contrast CT in PET–CT is still debated, the choice of high-dose PET–CT for the initial and final examinations, and low-dose imaging for the interim ones, may have influenced the results; however, without direct comparison of the two, no differences in diagnostic performance between high- and low-dose PET–CT were seen.

The combined use of PET–CT and EUS has high overall accuracy (90 per cent) in detecting recurrences in patients with upper GI malignancies resected with curative intent. In expert hands, EUS is superior to PET–CT in the detection of locoregional as well as isolated locoregional recurrence. Although PET–CT outperforms EUS in detecting distant metastases, the lack of survival benefit from treating disseminated recurrence with currently available modalities makes the clinical gain from this questionable.

## Funding

Danish Cancer Society

Fehr Foundation

Fionia Foundation

Region of Southern Denmark

University of Southern Denmark

## Supplementary Material

zraa028_Supplementary_Data

## Notes

Presented in part to the 12th International Hepato-Pancreato-Biliary Association World Congress, São Paolo, Brazil, April 2016
