# Supplementary material for: Value of regular endosonography and [18F]fluorodeoxyglucose PET–CT after surgery for gastro-oesophageal junction, stomach or pancreatic cancer
Source: BJS Open. 2020 Dec 23;5(2):zraa028. doi: 10.1093/bjsopen/zraa028 (PMC7944502; doi:10.1093/bjsopen/zraa028)
Supplement: zraa028_Supplementary_Data [file zraa028_supplementary_data.zip › zraa028_Supplementary_Data/Fig S1a-c figure legends incl.docx]

**Fig. S1a-c:** Cumulative diagnostic performance of PET/CT over time divided in sub-groups of a) Gastro-Oespohageal Junction, b) Stomach, and c) Pancreatic cancers.

a)


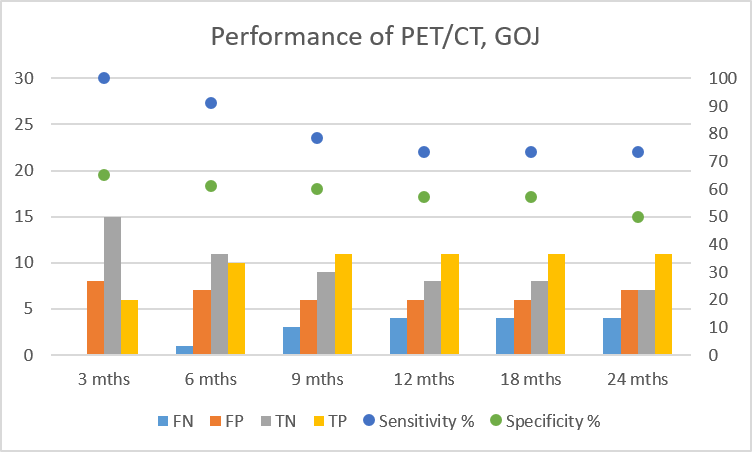


b)


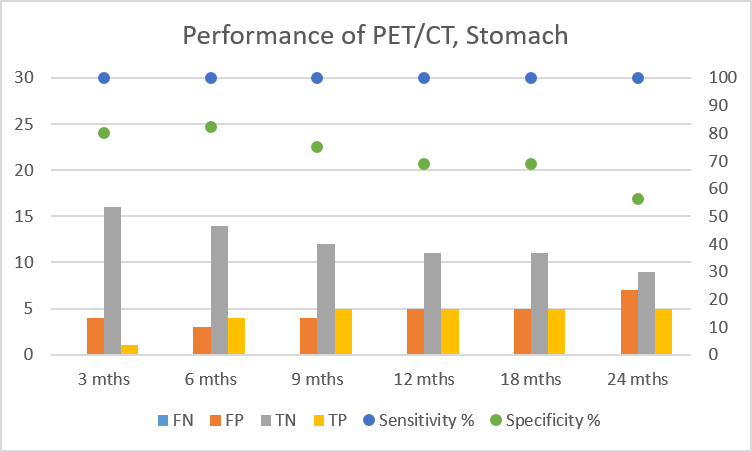


c)


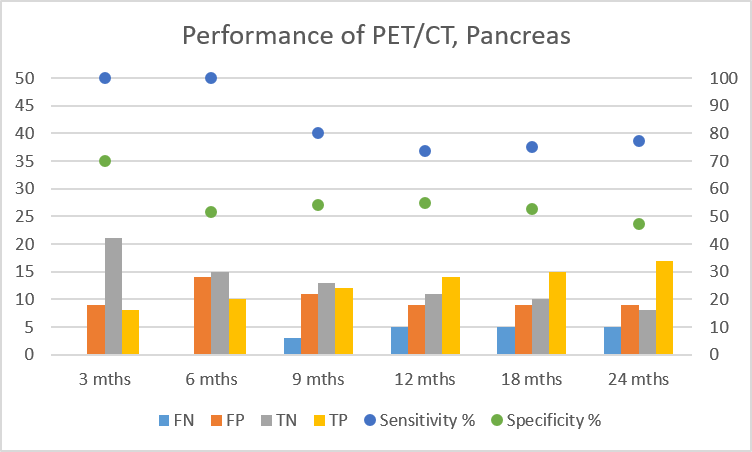


Abbreviations: PET/CT = positron emission tomography/computed tomography; FN = False negative; FP = False positive; TN = True negative; TP = True positive.
