# Supplementary material for: Value of regular endosonography and [18F]fluorodeoxyglucose PET–CT after surgery for gastro-oesophageal junction, stomach or pancreatic cancer
Source: BJS Open. 2020 Dec 23;5(2):zraa028. doi: 10.1093/bjsopen/zraa028 (PMC7944502; doi:10.1093/bjsopen/zraa028)
Supplement: zraa028_Supplementary_Data [file zraa028_supplementary_data.zip › zraa028_Supplementary_Data/Table S1 EUSvsPET.docx]

**Table S1** List and number of futile procedures due to false-positive PET/CT results.

| ***Procedure*** | ***Number*** |
| --- | --- |
| *Ultrasound guided biopsy* | 12 |
| *EUS-FNA* | 11 |
| *CT* | 5 |
| *Colonoscopy* | 5 |
| *MRI* | 4 |
| *Gastroscopy* | 3 |
| *PET/CT* | 2 |
| *Laparoscopy* | 1 |
| *Laryngoscopy* | 1 |

Abbreviations: EUS-FNA = Endoscopic ultrasound guided fine-needle aspiration; CT = computed tomography; MRI = magnetic resonance imaging; PET/CT = positron emission tomography/computed tomography.
