# Supplementary figures and images for: Value of regular endosonography and [18F]fluorodeoxyglucose PET–CT after surgery for gastro-oesophageal junction, stomach or pancreatic cancer
Source: BJS Open. 2020 Dec 23;5(2):zraa028. doi: 10.1093/bjsopen/zraa028 (PMC7944502; doi:10.1093/bjsopen/zraa028)

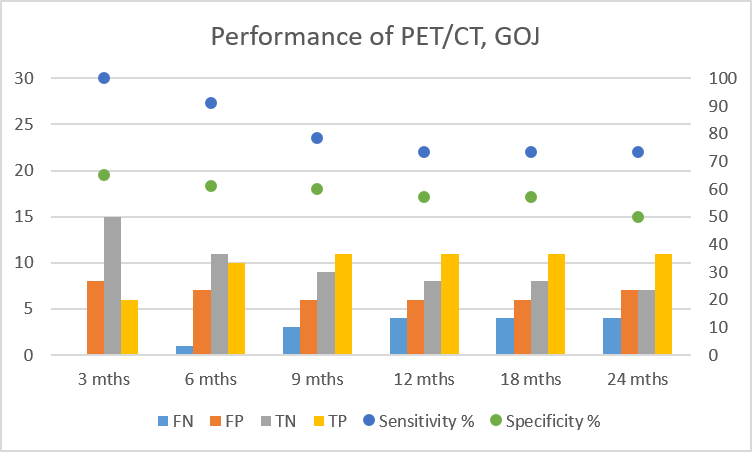

Supplement: zraa028_Supplementary_Data [file zraa028_supplementary_data.zip › zraa028_Supplementary_Data/Fig. S1a (GOJ) EUSvsPET.tif]

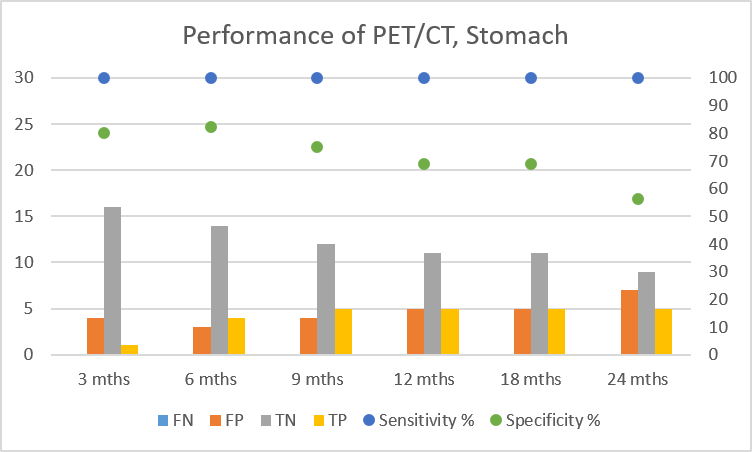

Supplement: zraa028_Supplementary_Data [file zraa028_supplementary_data.zip › zraa028_Supplementary_Data/Fig. S1b (Stomach) EUSvsPET.tif]

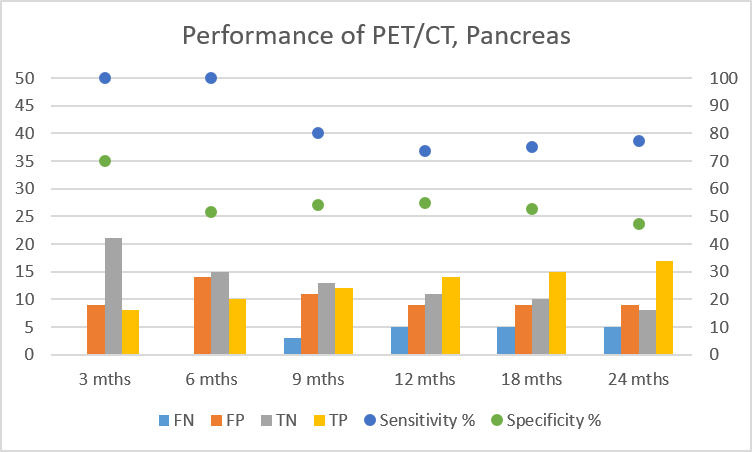

Supplement: zraa028_Supplementary_Data [file zraa028_supplementary_data.zip › zraa028_Supplementary_Data/Fig. S1c (Pancreas) EUSvsPET.tif]
